# Exercise-Induced Changes in Pulmonary Artery Stiffness in Pulmonary Hypertension

**DOI:** 10.3389/fphys.2019.00269

**Published:** 2019-04-02

**Authors:** Omid Forouzan, Eric Dinges, James R. Runo, Jonathan G. Keevil, Jens C. Eickhoff, Christopher Francois, Naomi C. Chesler

**Affiliations:** ^1^College of Engineering, University of Wisconsin–Madison, Madison, WI, United States; ^2^School of Medicine and Public Health, University of Wisconsin–Madison, Madison, WI, United States

**Keywords:** relative area change, pulse wave velocity, magnetic resonance imaging, exercise, stiffness and its variations

## Abstract

**Background:** Pulmonary hypertension causes pulmonary artery (PA) stiffening, which overloads the right ventricle (RV). Since symptoms of pulmonary hypertension (PH) are exacerbated by exercise, exercise-induced PA stiffening is relevant to cardiopulmonary status. Here, we sought to demonstrate the feasibility of using magnetic resonance imaging (MRI) for non-invasive assessment of exercise-induced changes in PA stiffness in patients with PH.

**Methods:** MRI was performed on 7 PH patients and 8 age-matched control subjects at rest and during exercise stress. Main pulmonary artery (MPA) relative area change (RAC) and pulse wave velocity (PWV) were measured from 2D-PC images. Invasive right heart catheterization (RHC) was performed on 5 of the PH patients in conjunction with exercise stress to measure MPA pressures and stiffness index (β).

**Results:** Heart rate and cardiac index (CI) were significantly increased with exercise in both groups. In controls, RAC decreased from 0.27 ± 0.05 at rest to 0.22 ± 0.06 with exercise (*P* < 0.05); a modest increase in PWV was not significant (*P* = 0.06). In PH patients, RAC decreased from 0.15 ± 0.02 to 0.11 ± 0.01 (*P* < 0.05) and PWV and β increased from 3.9 ± 0.54 m/s and 1.86 ± 0.12 at rest to 5.75 ± 0.70 m/s and 3.25 ± 0.26 with exercise (*P* < 0.05 for both), respectively. These results confirm increased MPA stiffness with exercise stress in both groups and the non-invasive metrics of MPA stiffness correlated well with β. Finally, as assessed by PWV but not RAC, PA stiffness of PH patients increased more than that of controls for comparable levels of moderate exercise.

**Conclusion:** These results demonstrate the feasibility of using MRI for non-invasive assessment of exercise-induced changes in MPA stiffness in a small, heterogeneous group of PH patients in a research context. Similar measurements in a larger cohort are required to investigate differences between PWV and RAC for estimation of MPA stiffness.

## Introduction

Pulmonary hypertension is a disease of abnormally high blood pressure in the vasculature of the lungs. The chronic increase in RV afterload in PH can suppress RV contractile performance and ultimately lead to RV dysfunction, which impairs exercise capacity and quality-of-life (Fourie et al., 1992; Sanz et al., 2009). Progression of RV dysfunction to RV failure is the cause of death in many PH patients. A major contributor to increased RV afterload is stiffening of the proximal PAs due to remodeling of the vessel wall (Tozzi et al., 1994; Wang and Chesler, 2013) and elevated distending pressures (Zuckerman et al., 1991; Lankhaar et al., 2006) and increased PA stiffness is associated with impaired RV performance (Stevens et al., 2012). Since functional symptoms of PH predominantly occur during exercise stress (Nootens et al., 1995; Lewis et al., 2013; Chaouat et al., 2014), studying the changes in main PA (MPA) stiffness in response to exercise stress could provide insight into exercise intolerance and RV dysfunction in PH. In addition, an abnormal pulmonary vascular response to exercise stress may enable earlier detection of disease.

The stiffness of a material is defined as the load required to cause deformation divided by the resulting deformation. In other words, a stiffer material requires more force to create the same deformation or has less deformation in response to the same force. The force (per unit area) on the PA is the transmural pressure; the deformation is its change in diameter or area. MPA stiffness can be measured invasively and estimated non-invasively. The gold standard measure of MPA stiffness is the stiffness index (β), which is calculated using systolic and diastolic pulmonary artery pressures obtained from right heart catheterization (RHC) and systolic and diastolic MPA diameters measured via imaging; increases in β predict disease progression (Hayashi et al., 1980; Sanz et al., 2009; Stevens et al., 2012; Tian and Chesler, 2012). However, as an invasive procedure, RHC incurs significant risk (Champion et al., 2009). Thus, non-invasively derived metrics of β offer significant advantages. Non-invasively derived metrics of MPA stiffness include the relative area change (RAC) and pulse wave velocity (PWV), both of which have been shown to predict outcomes in patients with PH (Gan et al., 2007; Swift et al., 2012). MRI is the gold-standard non-invasive technique for evaluating cardiopulmonary structure and function in PH (Gan et al., 2007). MRI is typically used at rest in clinical settings; however, recent developments in MRI-compatible exercise devices enable subjects to perform physical exercise inside MRI scanners (Forouzan et al., 2014). Thus, it is now possible to quantify non-invasive estimates of PA stiffness during exercise using MRI (Forouzan et al., 2015).

The objectives of this study were to investigate the feasibility of obtaining exercise-MRI derived metrics of β including RAC and PWV in patients with PH and to evaluate the correlation of RAC and PWV with β. We used MRI to measure RAC and PWV of MPA at rest and during exercise stress in 7 PH patients and 8 age-matched control subjects. 5 of the PH patients also performed exercise during RHC. With RHC-exercise and simultaneous MPA imaging by echocardiography, we computed β to compare the non-invasive and invasive metrics of MPA stiffness.

## Materials and Methods

### Subjects

This study was approved by the UW-Madison Institutional Review Board (#2011-0369 and # 2011-0890) and was compliant with the Health Insurance Portability and Accountability Act. A total of 7 patients with PH (6 females) and 8 control subjects (7 females) were recruited to perform exercise during MRI, following written informed consent and undergoing screening with MRI safety questionnaires. Enrollment criteria for PH patients were based on the pulmonologist referral; (1) adults (between the ages of 18 and 75 years) diagnosed with PH (2) NYHA functional class I, II, or III. Exclusion criteria were one or more of the following: (1) Recent syncope, (2) History of lung or cardiovascular diseases, (3) Severe skeletal or muscle abnormalities which could prohibit exercise, (4) Contraindication to cardiac MRI, (5) Presence of pacemaker or defibrillator in body, (6) Presence of metal in the body, (7) History of kidney disease, (8) Pregnancy or breastfeeding, (9) Vulnerable population (i.e., prisoners and those obviously lacking consent capacity), (10) Mixed etiology PH (e.g., IPAH, hypoxic PAH or any associated PAH and a history of isolated pulmonary emboli), (11) Severe lung disease: FEV1/FVC indicating severe obstruction; Total lung capacity <60%, (12) NYHA class IV patient. Table 1 summarizes patient information and hemodynamic measurements based on the clinical right heart catheterization for each patient. All control subjects were free of overt cardiovascular, pulmonary, and renal disease based on a self-report questionnaire. Five of the PH patients (4 females) also performed exercise under RHC, following written informed consent and under careful supervision of a physician.

**Table 1:** Pulmonary hypertension subject information and invasive hemodynamic measurements.

Subject	Sex	Age	NYHA	mPAP	PCWP
1	F	67	3	39	13
2	M	58	2	29	14
3	F	52	2	28	12
4	F	56	2	29	14
5	F	74	2	29	22
6	F	51	2	16	3
7	F	24	2	37	11


*NYHA, New York Heart Association; mPAP, mean pulmonary artery pressure; PCWP, pulmonary capillary wedge pressure; PVR, pulmonary vascular resistance. Units for pressure are mmHg and for PVR are dyne sec cm^-*5*^. Subject#6 was in remission from previously documented PH based on these results*.

### MRI-Data Acquisition and Procedure

Magnetic resonance imaging studies were performed on a clinical 1.5T scanner (GE Healthcare Optima MR450W, Waukesha, WI, United States) using an 8-channel cardiac coil and vector electrocardiographic (ECG) gating. Two-dimensional, phase contrast (2D-PC) images were acquired in double oblique planes through the MPA distal to the pulmonic valve. Image parameters for 2D-PC were: 35 × 26 cm field of view, supine position – head first, 256 × 160 acquisition matrix (reconstructed to 256 × 256), 7 mm slice thickness, ± 62.5 kHz bandwidth, 150 cm/s velocity encode (“venc”), TR/TE = 5.5/2.6 ms (full echo), prospective electrocardiographic gating with trigger delay of 10 ms, k-space segmentation factor of 8, and parallel imaging (ASSET) with an acceleration factor of 2. Images were reconstructed into 40 time frames throughout the cardiac cycle. Imaging was acquired at end-expiration and during an approximately 15∼17-s breath-hold.

To obtain blood flow velocity and CSA of MPA, 2D-PC images were analyzed by manually outlining contours of the MPA cross-section in the GE flow quantification software “CV Flow version 3.3” (GE Healthcare, Milwaukee, WI, United States). CO, or average MPA flow rate over one cardiac cycle, was calculated as the average product of the CSA and the flow velocity. CI was calculated as CO divided by BSA. SVI was calculated as the CI divided by the HR. RAC was calculated as [max CSA – min CSA/max CSA] (Gan et al., 2007). In the systemic circulation, PWV is typically calculated using the transit-time method, which is based on the travel-time of the flow waves between two specific locations separated by a known distance (Bradlow et al., 2007). Given the relatively short lengths of pulmonary arteries, the transit-time method is impractical in the pulmonary circulation. Therefore, the flow-area (QA) method was used here, in which PWV is calculated from the slope of the line fitted to the flow-area data (ΔQ/ΔA) during early systole (Vulliemoz et al., 2002). This method assumes a long wavelength relative to the vessel diameter and a linear Q-A relationship in early systole (Vulliemoz et al., 2002). To increase the temporal resolution of the MRI acquisition in this study, we used parallel imaging (ASSET) with an acceleration factor 2, which enhances the temporal resolution by two fold such that the true temporal resolution is 22 ms instead of 44 ms.

### MRI-Exercise Stress Procedure

Magnetic resonance imaging-exercise stress was conducted using a custom-made MRI-compatible device that allows patients to perform exercise with a leg-stepping motion while their torso is confined within the MRI bore (Forouzan et al., 2014). The device is equipped with an electronic sensor that measured stepping cadence real-time and a metronome that assists subjects in maintaining a set cadence. After acquiring images at rest, subjects performed moderate exercise (target workloads of∼35 W) of 3–4 min duration each. To capture consistent images and minimize the motion artifact, images were acquired during a 15∼20-s brief cessation from exercise.

### RHC Data Acquisition and Analysis

Right heart catheterization was performed using a standard Seldinger technique from the internal jugular vein approach. Pulmonary arterial pressure was measured using a Millar conductance catheter (Millar Instruments, Houston, TX, United States) and a properly zeroed pressure transducer. PA pressure was recorded continuously during exercise and simultaneously transthoracic Doppler echocardiography (Vivid 7 or Vivid E9, GE Healthcare, Waukesha, WI, United States) was used to measure MPA cross-sectional diameter through parasternal view. The continuous pressure signals for each patient were processed using a custom Matlab routine. For any signal containing amplitude variations between beats due to breathing, a polynomial fit was created from the data, subtracted from the original signal and then reset to the maximum systolic pressure in order to eliminate the underlying frequency. Following this, the various beats that made up the continuous pressure signal were averaged to provide a single, representative pressure waveform. The stiffness index (β) of the MPA (a dimensionless index) was calculated using PA pressures from RHC and RAC from simultaneous echo, as in Gan et al. (2007):

$$\text{β}\;=\;\frac{\text{1}}{\text{2RAC}}\text{Ln}\frac{\text{sPAP}}{\text{dPAP}}\;\;\;\;\;\;\;\;\;\;\;\;\;\;\;\;\;\;\;\;\;\;\;\;\;\;\;\;\;\;\;\left(1\right)$$

where sPAP and dPAP are the systolic and diastolic PA pressures, respectively.

### RHC-Exercise Stress Test

After data were acquired at rest, patients performed supine leg exercise on a bicycle ergometer (Medical Positioning Inc., Kansas City, MO, United States) mounted on the catheterization table. Beginning at an initial workload of 15 W, the workload was increased by 15 W every 2–4 min, while PA pressure and diameter and 12-lead ECG data were recorded simultaneously. Pressure data were continuously recorded during RHC. To reduce the discrepancy between MRI-exercise and RHC-exercise sessions, for each patient, only data that were obtained at RHC-exercise workloads in correspondence with MRI-exercise workloads were used in calculations.

### Statistical Analysis

All data are reported as mean value ± standard deviation (SD). A linear mixed effects model with subject specific random effects to account for repeated measurements (at rest and at exercise) was used to compare study outcomes between experimental conditions. Tukey’s honestly significant difference test was used as a *post hoc* test of significance. All *P*-values are two-sided and *P* < 0.05 was used to define statistical significance. Statistical analysis was conducted using SAS software (SAS Institute Inc., Cary, NC, United States) version 9.4.

## Results

Characteristics of the PH patients are displayed in Table 1. All but one PH patient had a resting mPAP greater than 25 mmHg. The one patient (#6, Table 1) with normal mPAP had previously been diagnosed as having PH but was in remission. We kept this subject in the PH group because the apparent duration of remission (time since last RHC) was less than the duration of PH (several years). Average age of control and PH subjects were 56 ± 16 and 55 ± 16 years, respectively. The etiology of PH in the majority of the patients (*n* = 6, 86%) was SSc-PAH. All study participants completed the exercise protocol and performed exercise during MRI without any problems, with no premature termination of tests. Control subjects and PH patients performed exercise at workloads of 32 ± 6 and 30 ± 8 W, respectively, and there was no significant difference between the exercise workloads of the two groups. There was no significant difference between the hemodynamic parameters (HR, SVI, and CI) of PH patients and control subjects at rest (Table 2). PH patients had lower RAC and higher PWV compared with controls at rest. The average mPAP at rest for PH patients was 33 ± 6 mmHg (Table 2).

**Table 2:** Summary of demographic information and characteristics at rest.

	Healthy controls (*n* = 8)	PH subjects (*n* = 7)	*P*-value
**Demographic information**		
Age (years)	56 ± 13.7	55 ± 15.8	0.89
Sex (n F/M)	7/8	6/7	0.93
BSA (m^2^)	1.92 ± 0.13	1.7 ± 0.15	<0.05
Diagnosis		SSc-PAH (6), CHD (1)	
NYHA (n ll/lll)		6/1	
**MRI at rest**			
HR (BPM)	69.63 ± 8.55	69.43 ± 17.15	0.98
CI (L/min)	3.31 ± 0.45	3.20 ± 0.77	0.76
SVI (ml)	47.86 ± 6.95	46.96 ± 9.67	0.84
RAC	0.27 ± 0.05	0.15 ± 0.02	<0.05
PWV (m/s)	2.25 ± 0.44	3.90 ± 0.54	<0.05
**RHC at rest (*n* = 5)**			
sPAP (mmHg)		50.00 ± 10.12	
dPAP (mmHg)		24.40 ± 4.83	
mPAP (mmHg)		32.93 ± 6.44	
β		1.86 ± 0.12	


*Data is presented as the mean ± SD. SSc-PAH, systemic sclerosis-pulmonary arterial hypertension; CHD, congenital heart disease; BSA, body surface area; NYHA, New York Heart Association; HR, heart rate; CI, cardiac index; SVI, stroke volume index; RAC, relative area change; PA PWV, pulmonary artery pulse wave velocity; sPAP, systolic pulmonary artery pressure; dPAP, diastolic pulmonary artery pressure; mPAP, mean pulmonary artery pressure. β, stiffness index coefficient*.

**FIGURE 1:**
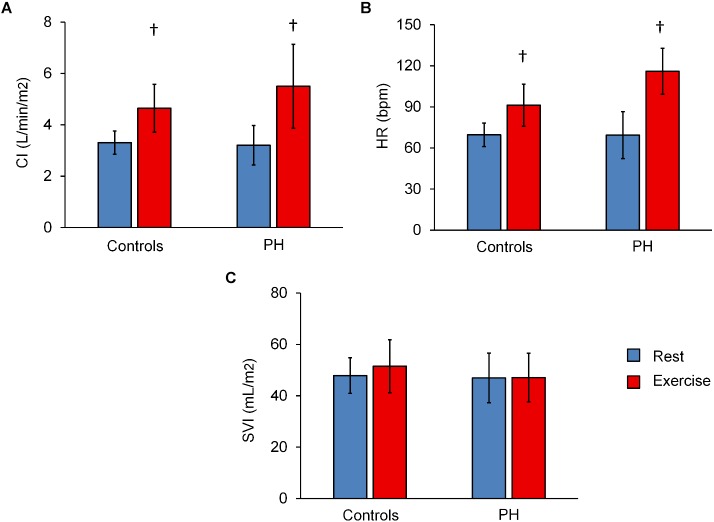
Summary of hemodynamics data at rest and with exercise. Averaged values of (CI) **(A)**, heart rate (HR) **(B)**, and stroke volume index **(C)** for control subjects and PH patients at rest and with exercise. Statistical analysis using two-way ANOVA with repeated measures shows an interaction effect between groups (controls vs. PH) and condition (rest vs. exercise) for HR (*P* = 0.04) but not CI or SVI. ^†^*P* < 0.05, vs. Rest. Mean ± SD shown.

CI and HR of both control subjects and PH patients significantly increased following exercise (Figure 1A,B). The increase in CI was not different between the control subjects and PH patients (interaction effect *P* > 0.05) but PH patients had a significantly greater increase in HR (70% in PH patients vs. 30% in controls subjects; interaction effect *P* < 0.05). SVI did not change with exercise for either group (Figure 1C).

**FIGURE 2:**
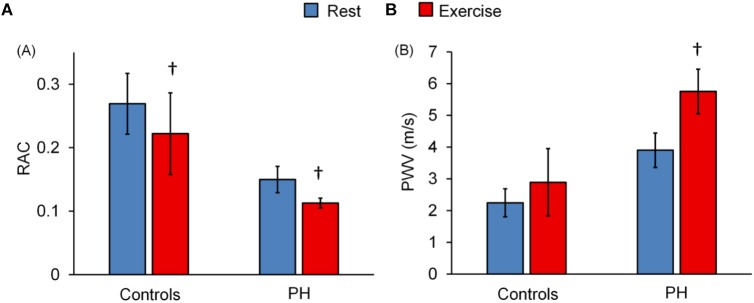
Effect of exercise on MPA-RAC and PWV. Lower RAC is associated with exercise for both control and PH subjects. PWV significantly increased with exercise only for PH patients. There is no interaction effect between the groups (control vs. PH) and conditions (rest vs. exercise) for RAC (*p* = 0.51), however, there was a significant interaction effect for PWV (*P* = 0.02). ^†^*P* < 0.05, vs. Rest. Mean ± SD shown.

Relative area change significantly decreased following exercise for control subjects and for PH patients whereas PWV significantly increased only in PH patients (Figure 2A,B). The decrease in RAC with exercise was not statistically different for control vs. PH subjects; in other words, there was no interaction effect between the two groups (controls vs. PH) with condition (Rest vs. Exercise). However, for PWV there was a significant interaction effect between controls and PH patients with condition (*P* < 0.05) indicating that patients had greater increase in PWV with exercise.

Five of the 7 PH patients also participated in a RHC-exercise procedure and performed exercise at 34 ± 7 W. With exercise, mPAP and pulse pressure (PP = sPAP – dPAP) significantly increased with exercise (Table 3). In two of the patients, PA diameter measured via echocardiography during the RHC-exercise procedure was not analyzable because of technical problems in acquisition. For the remaining PH patients, the stiffness index β increased 64% with exercise and this increase was significant (Table 3).

**Table 3:** Summary of invasive hemodynamics measured at rest and exercise in PH subjects.

	Rest	Exercise	*P*-value
mPAP (mmHg)	32.93 ± 10.12	44.73 ± 10.13	<0.05
PP (mmHg)	25.60 ± 6.11	37.00 ± 10.07	<0.05
β (*n* = 3)	1.86 ± 0.12	3.25 ± 0.26	<0.05


*Data is presented as the mean ± SD. mPAP, mean pulmonary artery pressure; PP, pulmonary artery pulse pressure (sPAP-dPAP); β, stiffness index coefficient*.

**FIGURE 3:**
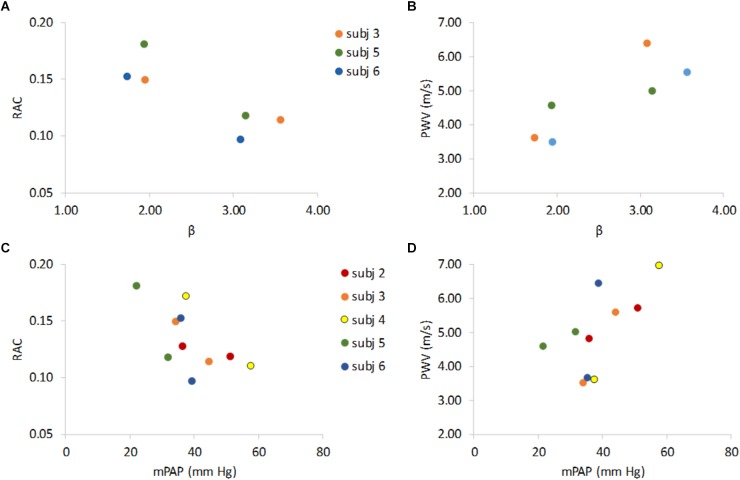
Non-invasive indices of MPA stiffness RAC and PWV versus invasive stiffness index (β) and vs. mean pulmonary artery pressure (mPAP). RAC vs. β and PWV vs. β show that lower RAC is associated with higher β **(A)** and higher PWV is associated higher β **(B)**. RAC and PWV vs. mPAP show that an increase in mPAP associates with lower RAC **(C)**, and higher PWV **(D)**. Data are color-coded by subject with rest data at lower β (panels **A,B**) or lower mPAP (panels **C,D**) for 5 the PH subjects that underwent both RHC and MRI and the 3 PH subjects for whom β could be calculated at both rest and exercise.

As expected, decreased RAC and increased PWV were associated with increased β (Figure 3A,B) and RAC decreased and PWV increased with increased mPAP (Figure 3C,D).

## Discussion

The main findings of this study are that non-invasive metrics of MPA stiffness measured with MRI increase during moderate exercise stress in PH patients and there was larger stiffness increase in patients compared with controls as assessed by the pulse wave velocity. We also found that with exercise both mPAP and PA stiffness increased. Since functional symptoms of PH predominantly occur during exercise stress (Nootens et al., 1995; Lewis et al., 2013; Chaouat et al., 2014), increases in PA stiffness in response to exercise may provide insight into exercise intolerance and RV dysfunction in PH. The ability to identify an abnormal pulmonary vascular response to exercise stress may also lead to earlier diagnosis of pulmonary vascular disease.

The cardiac stress test, which is an important diagnostic and prognostic tool in evaluating the cardiovascular status, can be conducted via pharmacological stress or physical exercise stress (Argiento et al., 2010; Sharma et al., 2015). However, the cardiovascular stress induced by physical exercise better represents conditions of daily life (Armstrong and Zoghbi, 2005). Exercise stress is typically performed with echocardiography. Since MRI is the gold-standard non-invasive method for assessing RV function (Lorenz et al., 1995; Pennell and Casolo, 1997), and RV failure is the most frequent cause of death in PH patients, there is increasing interest in using exercise with MRI for PH patients. Recently, exercise-MR was combined with invasive measurements of PA pressure (Claessen et al., 2015), but this is not possible at most institutions and is unlikely to become a clinical standard of care. Here, we used a custom-made MRI-compatible device (Forouzan et al., 2014) to conduct physical exercise within the MRI scanner. Our results indicate that HR and CI of both controls and PH patients increased with exercise, which demonstrates that the exercise workloads were effective in inducing cardiovascular stress. We found no change in SVI in response to exercise, which is consistent with supine exercise since the supine position increases venous return and stroke volume and no additional increase occurs during exercise (Elstad et al., 2009).

To estimate MPA stiffness non-invasively, we used the RAC, which is more accurately a measure of area strain. Unlike true stiffness metrics such as β, which require measures of load and deformation, RAC can be calculated from deformation alone. Decreased RAC is correlated with poor prognosis in PH patients (Gan et al., 2007; Swift et al., 2012). PWV, which measures the propagation velocity of the pulsatile pressure wave, is also useful in assessing the elastic properties of the MPA (Milnor et al., 1969; Ibrahim el et al., 2011). For linearly elastic cylinders, PWV is equal to the square root of the elastic modulus times the wall thickness divided by the diameter of the cylinder and the density of the fluid. Thus, PWV is higher for stiffer and smaller arteries. Our results confirm prior studies showing that the MPA of PH patients deforms less than that of control subjects; that is, RAC is lower in PH patients. Here, we further show that the MPA stiffness of both control subjects and PH patients increases with exercise. Moreover, when PWV is used to estimate MPA stiffness, the response of PH patients to exercise is significantly different from the response of controls. This novel finding, that the MPA PWV of PH patients increases more than that of controls for comparable levels of moderate exercise and comparable increases in CI, was evident despite a despite a small number of subjects, which indicates a large effect size.

Arterial stiffening is often described as an irreversible conformational change that progresses with the disease and is caused by collagen accumulation and wall thickening (D’Alonzo et al., 1991; Hyduk et al., 2005; Kobs and Chesler, 2006). However, arteries can acutely become stiffer due to wall stretch (Fung, 1993) or an acute increase in pressure (Zuckerman et al., 1991; Bellofiore et al., 2013), both of which cause reversible conformational changes in the vessel wall structure, such as collagen uncrimping and extension. It is not yet clear whether the increased PA stiffness observed in PH is only due to pressure-independent structural changes in the vascular wall as demonstrated in preclinical studies (Handoko et al., 2009; Kobs and Chesler, 2006; Ooi et al., 2010) or is also an effect of elevated distending pressures (Mereles et al., 2006; Kowal-Bielecka et al., 2008; Bellofiore et al., 2013). The strong association we found between MPA stiffness and mPAP, and the increase in both with exercise stress, support the suggestion that a significant contributor to MPA stiffness is increased mPAP. Also, the larger increase in PWV in PH patients in response to exercise may be due to the larger increase in mPAP. Future studies in a larger patient population are required to confirm the ability of non-invasive metrics of MPA stiffness to serve as non-invasive indicators of mPAP and thus PH severity.

The non-invasive stiffness parameters RAC and PWV were moderately associated with the RHC-derived stiffness β. Previously, correlations between these metrics have been shown at rest (Gan et al., 2007). Our results show that the correlations also exist during exercise, which supports the claim that these non-invasive stiffness metrics are useful indicators of cardiopulmonary status that can be used at different functional states and for a wide range of physiological conditions.

An important identifier of RV dysfunction in PH patients is diminished ventricular-vascular coupling, which represents the inability of RV contractility to match the increased afterload (Bellofiore et al., 2013). Decreases in ventricular-vascular coupling with exercise were recently reported in PH patients (Spruijt et al., 2015). Since PA stiffness is an important component of the RV afterload, studying the changes in PA stiffness in response to exercise stress could provide insight into impaired ventricular-vascular coupling with exercise.

There are several limitations to this study. First, the MRI-exercise and RHC-exercise procedures were performed on different days, which could potentially affect the accuracy of direct comparisons. Due to the small sample size, our novel results require confirmation by larger studies. The small study population also precludes subpopulation analyses by PH subtype. Another limitation is that subjects did not perform maximal exercise workloads. This remains a challenge within MRI scanners since there is restricted range of motion. While the effectiveness of our exercise protocol in inducing cardiac stress was confirmed by significant increases in HR and CI in both study groups, assessing hemodynamic responses at higher workloads and especially closer to maximal exercise workloads could provide additional insight into the functional state of the cardiopulmonary system.

## Conclusion

In the present study, we demonstrated the feasibility of using MRI exercise-stress to measure MPA stiffness in PH patients. Our results confirm that MPA stiffness increases with exercise in both control subjects and PH patients and that PH patients have stiffer MPA at rest compared with controls. Moreover, despite a small sample size, our results show that PWV increases more in PH patients than in controls with exercise. From a clinical perspective, using MRI-exercise stress for non-invasive measurement of arterial stiffness could be a feasible and practical option for frequent monitoring of PH progression.

## Ethics statement

All procedures were approved by UW-Madison Health Sciences Institutional Review Board.

## Author Contributions

OF participated in the study design, contributed to study coordination and data acquisition, performed the quantitative data analysis, interpreted data, and drafted the manuscript. ED contributed to study coordination and data acquisition, and performed the quantitative data analysis. CF, JR, and JK contributed to the study design and preparation of the manuscript, and supervised the data collection. JE contributed to data analysis and preparation of the manuscript. NC conceptualized the study objectives, obtained funding, and contributed to the study design, interpretation of data, and preparation of the manuscript.

## Conflict of Interest Statement

The authors declare that the research was conducted in the absence of any commercial or financial relationships that could be construed as a potential conflict of interest.

## Abbreviations

β: stiffness index coefficient
2D-PC: two-dimensional phase-contrast
BSA: body surface area
CHD: congenital heart disease
CI: cardiac index
CO: cardiac output
CSA: cross-sectional area
dPAP: diastolic pulmonary artery pressure
HR: heart rate
MPA: main pulmonary artery
mPAP: mean pulmonary artery pressure
MRI: magnetic resonance imaging
PA: pulmonary artery
PH: pulmonary hypertension
PP: pulmonary artery pulse pressure
PWV: pulse wave velocity
RAC: relative area change
RHC: right heart catheterization
RV: right ventricular
sPAP: systolic pulmonary artery pressure
SSc-PAH: systemic sclerosis pulmonary arterial hypertension
SV: stroke volume
SVI: stroke volume index
